# Molecular Modeling of Histamine Receptors—Recent Advances in Drug Discovery

**DOI:** 10.3390/molecules26061778

**Published:** 2021-03-22

**Authors:** Pakhuri Mehta, Przemysław Miszta, Sławomir Filipek

**Affiliations:** Biological and Chemical Research Centre, Faculty of Chemistry, University of Warsaw, 02-093 Warsaw, Poland or pakhurimehta@gmail.com (P.M.); pmiszta@chem.uw.edu.pl (P.M.)

**Keywords:** histamine receptors, G protein-coupled receptors, computational studies, molecular docking, virtual screening, drug discovery and design

## Abstract

The recent developments of fast reliable docking, virtual screening and other algorithms gave rise to discovery of many novel ligands of histamine receptors that could be used for treatment of allergic inflammatory disorders, central nervous system pathologies, pain, cancer and obesity. Furthermore, the pharmacological profiles of ligands clearly indicate that these receptors may be considered as targets not only for selective but also for multi-target drugs that could be used for treatment of complex disorders such as Alzheimer’s disease. Therefore, analysis of protein-ligand recognition in the binding site of histamine receptors and also other molecular targets has become a valuable tool in drug design toolkit. This review covers the period 2014–2020 in the field of theoretical investigations of histamine receptors mostly based on molecular modeling as well as the experimental characterization of novel ligands of these receptors.

## 1. Introduction

The most recent scientific technologies have crucial applications for drug discovery and design [1,2,3,4]. In silico approaches such as virtual screening and molecular docking have been widely applied to diverse proteins including G protein-coupled receptors (GPCRs) which constitute the largest family of cell surface receptors in the human body comprising, among others, histamine, dopamine, adenosine or adrenergic receptors that play a key role in cellular signaling [5,6,7,8]. Developments in GPCR structural biology provide insights into GPCR-ligand binding. In the recent years, a tremendous progress has been made in the crystallization of GPCRs representing different GPCR families and subfamilies, including aminergic GPCRs such as the histamine H_1_ receptor (H_1_R). Crystallographic information concerning GPCRs is essential for understanding the possible ligand-protein interactions and diverse conformational changes associated with multiple downstream signaling paths. Obtaining crystallographic data for GPCRs has been a very difficult task due to their conformational flexibility and heterogeneity of this superfamily. The crystal structures of GPCRs proved to be very useful for structure-based ligand design methods, screening of compound libraries and building homology models of not yet crystallized GPCRs [5,6,9,10]. With the crystal structure of H_1_R, a new opportunity has emerged to prepare the homology models of other histamine receptor subtypes (H_2_R, H_3_R and H_4_R) together with a combination of ligand-based and structure-based drug design [11,12]. Structural information provided by H_1_R as well as adrenergic β_2_-AR and other templates was used to resolve the structures of HRs in the inactive and active states in order to understand GPCR functionality and generate more effective drug discovery strategies. Recent investigations on the role of HRs in (patho)physiology and the use of receptor antagonists in in vivo disease models reveal a vast potential of histamine receptors in the treatment of e.g., allergic inflammation, neuropathic pain, and cancer [13,14,15,16,17,18,19,20,21]. The search for new and potent HR antagonists contributes to a steadily increasing number of potent and structurally diverse compounds [6,22,23,24,25,26,27,28]. Furthermore, development of ligands that are able to bind to two or more HR subtypes offers another opportunity to achieve a synergistic clinical effect mostly for allergic inflammations and neuropsychiatric disorders [29,30,31,32,33]. Taken together, the HRs field is showing a lot of potential to deliver another generation of potent drugs for clinical studies. In this review, we focus on the applicability of molecular modeling including drug discovery and design procedures such as molecular docking and virtual screening performed on HRs. We also review relevant clinical candidates and their therapeutic potential for the treatment of various inflammatory and allergic diseases.

### 1.1. Overall Structure of the Histamine Receptors

Histamine receptors, which belong to class A of the GPCR superfamily contain a bundle of seven antiparallel transmembrane helices, TM1 to TM7, connected by three extracellular loops, ECL1 to ECL3, and three intracellular loops, ICL1 to ICL3. Some helices contain highly conserved sequence motifs which are necessary to conduct activation steps. Furthermore, in all helices there are evolutionarily conserved residues required to preserve proper structure and function of GPCRs. Those residues are denoted by the number “50” in each helix in the Ballesteros-Weinstein numbering scheme: N^1.50^, D^2.50^, R^3.50^, W^4.50^, P^5.50^, P^6.50^ and P^7.50^ [34,35,36]. In this numbering scheme each residue of GPCR is recognized by two numbers separated by a dot; the first number indicates the transmembrane helix while the second number the position of the residue relative to the most conserved residue (assigned the number 50) on the same helix in a sequential order [34]. The most recognized sequence motifs in class A GPCRs are associated with molecular switches existing also in HRs; they are: the ionic lock associated with the DR^3.50^(Y/F) motif in TM3, the transmission switch in TM6 associated with the CWxP^6.50^ motif, and the tyrosine toggle switch in TM7 (NP^7.50^xxY motif) [37,38]. In the H_1_R receptor crystal structure the classical ionic lock is absent and R^3.50^, instead forming a salt bridge with a negatively charged residue in TM6, forms a hydrogen bond with Q^6.36^. The hydrogen bond can also link helices TM3 and TM6 but lack of the ionic interaction is intriguing and can modulate the activation of HRs. Since the activated structure of histamine receptors has not been determined the details of the activation steps remain unknown. Nonetheless, this structure must be similar to that of other amine-activated GPCRs, since some residues typical for this group, such as D^3.32^ and W^7.40^, are also present in all HRs [39]. D^3.32^ is directly bound to the ligand, while W^7.40^ is not in contact with the ligand but is located just behind the 3–7 lock [37,38] indicating importance of this molecular switch for activation of amine-activated receptors. Apart from differences in the ligand binding sites there are also other differences in a sequence and the average sequence identity between HR subtypes is only about 20–30% [11]. HRs are divided into four subtypes, H_1_R-H_4_R, that present also some differences related to tissue expression, ligand specificity and the final cellular effects.

Since the publication of the first crystal structure of GPCR describing the 3D structure of rhodopsin in 2000, multiple GPCRs have been crystallized including one histamine receptor [40]. The structure-based drug design using HR homology modeling has only been made possible after determination of the 3D-structure of bovine rhodopsin. The crystal structure of the histamine H_1_ receptor was obtained in 2011 and is still the only structure of histaminergic receptor deposited in the Protein Data Bank [41]. The structure of H_1_R provided important insights into the ligand binding mode in HRs and was successfully applied for drug discovery and design purposes [42]. The structures of GPCRs greatly contributed to and continue to provide great opportunities for the discovery and design of novel ligands using the structure-based approaches [43,44,45].

### 1.2. Recent Publications on Computational Studies Targeting HRs

**Figure 1:**
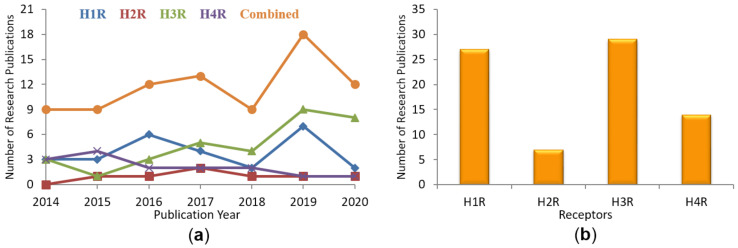
Number of histamine (H_1_–H_4_) receptor-related computational publications in the 2014–2020 time period: (**a**) Yearly on each HR; (**b**) Total publications on each HR.

The research papers describing in-silico approaches applied for the discovery of novel ligand chemotypes targeting HRs published in the recent years (2014–2020) have been compiled in Figure 1. These research papers were located in PubMed, Google Scholar and SciFinder using the expressions “histamine receptor docking” and “histamine receptor virtual screening”, and then filtering out those papers which did not directly deal with HRs. This search yielded over 80 novel research papers. Figure 1a depicts the number of research papers published yearly on each receptor as well as the number of publications covering all HRs. It can be observed that the number of research publications in the histamine H_3_R field shows an increasing trend after 2015 while the year 2019 saw a boom in the number of drug discovery projects in HR field with the exception of H_2_R and H_4_R. In 2020 the coronavirus pandemic was probably a direct reason for a much smaller number of papers. Figure 1b depicts the total number of papers published on each HR in the 2014–2020 time period. In the following sections, we review the most recent research publications describing computational approaches applied for the discovery of novel HR ligands and residues responsible for ligand binding and receptor activity.

Recently, Zobayer and Hossain studied the physicochemical and structural properties of HRs. The 3D models of HRs were developed through the homology modeling methodology using the I-TASSER webserver and the best model for each receptor was selected by applying various structure-validation tools [46]. The homology models of all GPCRs were created and made available in the GPCRdb webserver which is the information hub for GPCRs and their complexes [47]. The availability of the crystal structure of H_1_R, along with the homology models of other HRs, have resulted in the development of HRs-targeted ligands exemplified in Table 1. The crystal structure of H_1_R provides the highest hit rates while the hit rates for homology models vary to some extent.

## 2. HR-Targeted Ligands and Receptor Binding Site of Inactive/Active States of HRs

**Figure 2:**
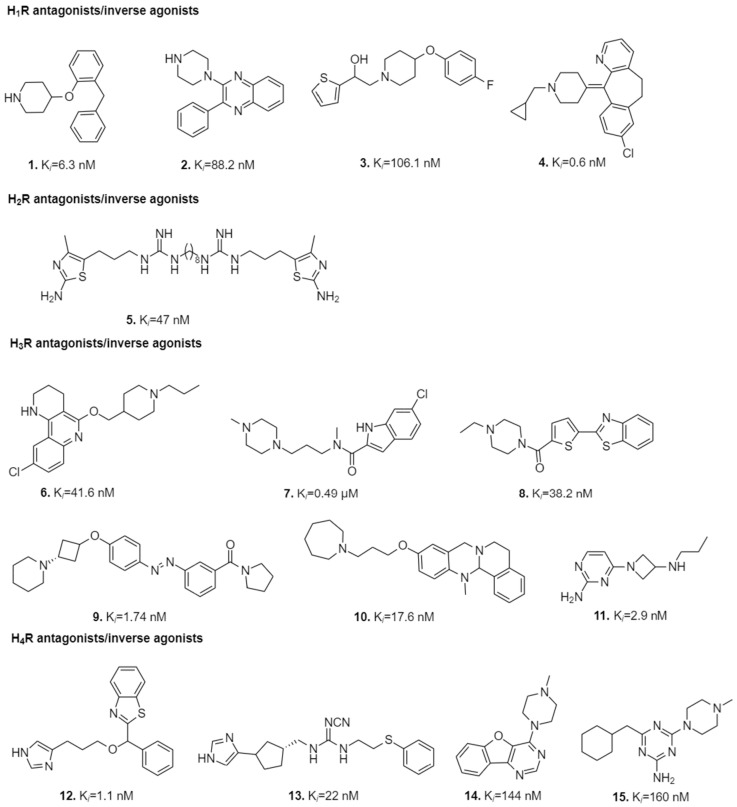
The most potent ligands targeting HRs discovered through diverse virtual screening and docking approaches in 2014–2020 and their biological activities.

Virtual screenings and molecular docking have led the path towards development of novel HR-targeted ligands with high affinities for particular subtypes of HRs (Figure 2). The novel ligands display predicted interactions with crucial residues in the binding sites of HRs. In Table 2 these residues are presented with their original numbers from particular receptors and also in B-W numbering scheme.

### 2.1. Computational Studies on Histamine H_1_ Receptor and Its ligands

#### 2.1.1. Structural Aspects of Histamine H_1_ Receptor

**Figure 3:**
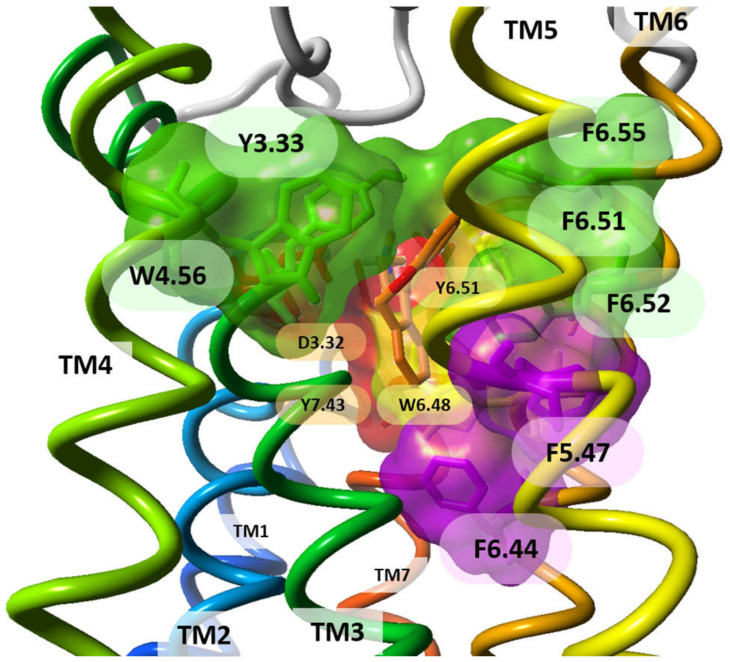
The areas of amino acids surrounding doxepin in the crystal structure of H_1_R: (red) the amine-binding region; (green) the upper aromatic region; (purple) the lower aromatic region; (yellow) the boundary amino acids participating in both amine-binding and aromatic regions. Doxepin is shown with its carbon atoms colored in orange. Side view of the receptor. TM helices are colored from blue (TM1) to red (TM7).

The first HR deposited in Protein Data Bank in 2011 was H_1_R in an inactive conformational state (PDB id:3RZE) [41]. The orthosteric binding site of this receptor is confined between the upper regions of the transmembrane helices (TMHs) and the extracellular loops (ECLs). H_1_R (UniProt id:P35367, 487 amino acids) crystal structure has a disulphide bond linking C180 (ECL2) with the extracellular end of TM3 (C^3.25^) but it is lacking the palmitoylation site at the end of helix H8 which is present in many other GPCRs. The antagonist orthosteric binding site in the H_1_R structure is divided into three regions lined by crucial residues as suggested by several site-directed mutagenesis studies: (i) the amine-binding region (D^3.32^, W^6.48^, Y^6.51^, I^7.39^, and Y^7.43^); (ii) the upper aromatic region (Y^3.33^, W^4.56^, Y^6.51^, F^6.52^, and F^6.55^), and (iii) the lower aromatic region (F^5.47^, F^6.44^, and W^6.48^), positioned deep in the TMHs [72] (Figure 3). The first-generation H_1_R antagonist doxepin sits deep in the ligand-binding pocket and directly interacts with W^6.48^, a highly conserved key residue required for GPCR activation, which is stabilized upon antagonist binding. The amine moiety of doxepin interacts with residue D^3.32^ present in all aminergic GPCRs. Both the upper and lower aromatic regions accommodate the butterfly-shaped hydrophobic aromatic moieties of doxepin. All these three regions were investigated to unravel H_1_R molecular determinants and visualize the binding hotspots in order to determine high affinity H_1_R ligands. The combined WaterFLAP calculations (approach to predict the binding site waters to guide ligand docking) and site-directed mutagenesis studies emphasized the crucial role of residue 7.39 (Table 2), a highly variable residue in aminergic GPCRs, as a determinant of specific N-methyl effects in amine ligand binding and responsible for stereo- and subtype-selectivity [72]. The second generation H_1_R antagonists containing unique carboxyl groups interacted with K^5.39^ and/or K179^ECL2^ in the anion-binding region of H_1_R leading to improved receptor pharmacology. This region is not conserved in other aminergic receptors illuminating the molecular basis of H_1_R antagonistic specificity and selectivity. The TM4 of the aminergic histamine H_1_R is also constricted, directing W^4.56^ (an important residue for H_1_R-ligand binding, based on mutation studies) towards the aromatic ligand binding pocket.

#### 2.1.2. H_1_R Targeted Ligands and Their Interactions in the Ligand-Binding Pocket

##### Receptor-Based in Silico Approaches Targeting H_1_R

Several researchers have pioneered in the field of discovery of HR antagonists, especially those of H_1_R, as its crystal structure has been available since 2011. Both mutagenesis and computational studies indicated the importance of interactions with D^3.32^, Y^3.33^, T^5.42^, N^5.46^, W^6.48^, Y^6.51^, F^6.52^, and F^6.55^ in the orthosteric pocket for histamine and doxepin binding, and underlined the crucial electrostatic interaction with the side chain of D^3.32^ [75]. The other residues present in H_1_R and bound to the ligand were found to be K179^ECL2^, K^5.39^, H^7.35^ and Y^7.43^ [56]. Multiple walker metadynamics-simulation protocol was used for the identification of the preferential binding mode of the physiological ligand histamine obtained from 92% of conformational ensembles [99]. Pose re-scoring of doxepin at H_1_R followed by multiple linear regression using Prime software of Schrodinger and MD simulations proved to be essential for predictive modeling of receptor-ligand interactions [84]. Enrichments in virtual screenings have been improved using Interaction Fingerprints (IFPs) such as the SYBYL software capturing atom–atom interactions and SPLIF (Structural Protein-Ligand Interaction Fingerprints) capturing fragment–fragment co-occurrences [5]. Also, the virtual screenings of fragment-like compounds, using consensus energy-based docking scoring approach including both IFP (≥0.75) and PLANTS (≤−90), and the ionic interaction with residue D^3.32^ used as a filter proved to be better than individual scoring algorithms with increased hit rate of 73%. These attempts led to the efficient identification of chemically novel H_1_R antagonists (ECFP-4 similarity cut-off of 0.4) with nanomolar affinities and potencies such as Compounds **1**–**3** (Figure 2) [9,72].

**Figure 4:**
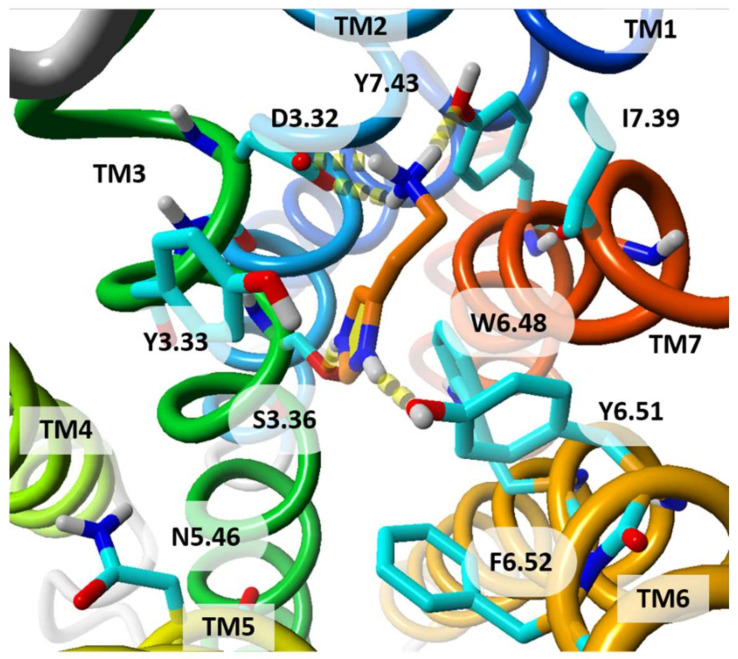
Histamine molecule docked to the crystal structure of hH_1_R. The histamine is doubly positively charged. The amine group is bound to D^3.32^ and Y^7.43^, and the imidazole ring to S^3.36^ and Y^6.51^. The residue numbers are shown in Ballesteros-Weinstein numbering scheme. Histamine is shown with its carbon atoms colored in orange. View from extracellular side. TM helices are colored from blue (TM1) to red (TM7).

To demonstrate differences in the orthosteric binding sites of histamine receptors we have docked doubly protonated histamine to explore strong binding modes. The protonation state and the chosen pose of histamine are used for illustrative purposes only. The ligand histamine has been selected since the histamine molecule is small, so it does not change the binding site much, and is able to bind to all histamine receptors. The most likely binding mode of doubly protonated histamine in hH_1_R is shown in Figure 4.

Diverse multi-target compounds, including indolecarboxamides and alkyl/aryl piperidyl indoles, were reported as H_1_R, serotonin-5HT and CCR2 antagonists [65,69] while phenothiazines (promethazine, chlorpromazine, 2-chlorophenothiazine, thioridazine, trifluoperazine) were reported to be MRGPRX2 activators and H_1_R antagonists [100]. Chlorpromazine has high affinity to other GPCRs including dopamine, norepinephrine and muscarinic receptors [101,102]. The polypharmacology effect [103,104] of such ligands highlights the importance of other GPCRs, not only H_1_R, as potential templates for homology modeling of HRs. Indolecarboxamides were reported to form hydrogen bonds with N443^ECL3^, R176^ECL2^, and I^6.58^ [65] while alkyl/aryl piperidyl indole formed electrostatic interactions with conserved Y^6.51^ residue [69]. Several other compounds have been docked to the H_1_R structure such as aminomethylenepyrimidine-2,4,6-triones, N^1^-alkyltheobromine, N-methylanthranilates, azabicyclic isoxazoline acylhydrazones, substituted tetrazole-incorporated quinoline derivative, synthesized rupatadine and desloratadine analogues [70,73,76,105]. Elbayaa [70] designed a series of substituted aminomethylenepyrimidine-2,4,6-trione derivatives generated from a four-featured pharmacophore model with an aromatic or π-ring system, hydrophobic group, a H-bond donor and a H-bond acceptor group derived from five H_1_R antagonists and validated this model using six other H_1_R antagonists. These antagonists were mapped on the pharmacophore model with a good fitting score and low RMSD and then were docked using Molegro Virtual Docker on the H_1_R model obtained from SWISS-MODEL. They were reported to be a promising template for designing novel non-sedating H_1_R antihistaminic agents [70]. Docking studies, using AutoDock, showed that derivatives of N^1^-alkyltheobromine, which exhibited H_1_R antihistaminic activity comparable to doxepin and less CNS depressant side effects than olapatadine, interacted electrostatically and formed hydrogen bonds with residues D^3.32^, Y^3.33^, S^3.36^, T^3.37^, K^5.39^ and Y^6.51^ [71]. In one study, quinoline derivative (QS-15), astemizole and diclofenac sodium have been shown to interact with residues D^3.32^, W^6.48^ and F^6.52^ [73]. Additionally, although the fragment VUF13816 is structurally different from the reported H_1_R ligands obtained from virtual screening, it was observed to have similar contacts to the receptor as doxepin (residues H^7.35^ and D^3.32^). A series of three fluorescent ligands was designed based on this small fragment that retained similar affinity towards H_1_R as the parent compound [74].

In another study [55], fexofenadine, a potent non-sedative third-generation hH_1_R antagonist was proved to be beneficial in treating H_1_R related allergic conditions of dogs and cats. In this study, the homology models of dog and cat H_1_R isoforms were built and fexofenadine was subsequently docked to human, dog and cat H_1_R. In total, it interacted with 23 residues in all the three receptors, and the most crucial molecular determinants in hH_1_R were W^6.48^, F^6.52^, Y^3.33^, N^5.46^ and T^5.42^ [55]. The potential phytochemical inhibitors targeting both H_1_R and cytosolic phospholipase A_2_, with good pharmaceutical drug-like properties, such as 3′,4′,7-trihydroxyflavone, calycosin, geraldone, licoflavanone and epidistenin were reported to interact with D^3.32^, S^3.36^, Y^6.51^, H^7.35^ as well as T182^ECL2^ and D183^ECL2^ [48]. Also, the anti-allergic and anti-inflammatory potential of phytocomponents was analyzed by computational docking analysis on H_1_R [87,97,106]. The examined compounds included: β-pinene, thymol and carvacrol, present in the Siddha formulation *Oma Legium*, the standard cetrizine and bioactive phytotherapeutics such as ascorbic acid, β-sitosterol, sesquiterpene, and tocopherolpresent in the medicinal herb *Corallocarpus epigaeus*, as well as flavones like kaempferol and kaempferol-3-glucuronide found in *Centratherum punctatum* along with desloratadine as a reference standard. The flavones and desloratadine showed similar ligand-protein interactions with residues Y^6.51^ and F^6.52^ [87]. Curcumin has also been shown to possess H_1_R antagonistic activity [56]. About 22 synthesized rupatadine and desloratadine analogues, including Compound **4** (Figure 2), were docked to H_1_R and, guided by docking studies, the steric constraints within the binding pocket were found to explain the observed differences in affinity of ligands. The limiting residues were I^7.39^ and Y^7.43^ located next to the amine-binding region [98]. Methyl (MMA), propyl (PMA) and isopropyl (IMA) N-methylanthranilate, originally found in the leaf essential oil of *Choisya ternata*, were reported to establish interactions with D^3.32^, Y^3.33^, S^3.36^, I^3.40^, W^4.56^, N^5.46^, F^5.47^, F^6.44^, W^6.48^, Y^6.51^, F^6.52^, F^6.55^ and I^7.39^ [76]. In 2020, a set of 35 antihistamines was designed using cloperastine as the core molecule in docking and molecular dynamics studies, however, no experimental binding studies were performed [107]. Another study involving in silico design, synthesis, ADME profiling and evaluation of antagonistic effects of 1,8-naphthyridine-3-carboxylic acid analogues was carried out using chlorpheniramine as the standard drug. Ligand dockings using Auto Dock Vina elucidated the crucial interactions in the binding pocket of H_1_R, involving residues D^3.32^, Y^3.33^, S^3.36^ and Y^6.51^, for the ligands possessing satisfactory ADME profiles [108].

##### Ligand-Based Computational Approaches in Search for Potential H_1_R Ligands

Fragment-based drug discovery proved to be a propitious approach for the development of novel chemically and therapeutically active leads. The identification of small fragment-like biologically active dual H_1_R/H_4_R antagonists has been made possible through prospective ligand-based virtual screening (LBVS) using 14 different chemical similarity descriptors and consensus scoring approaches [27]. It was evident through this study that the performance of the similarity descriptors decreases with decreasing self-similarity of the actives. Also, the Molecular ACCess System (MACCS) turned out to be one of the three best performing similarity descriptors for H_1_R and H_4_R, while piDAPH3 and piDAPH4 being the worst. Certain consensus scoring methods achieve better enrichments, with best results in both the max-value or ranked-by-vote consensus methods as became evident in study of H_1_R and H_4_R virtual screening enrichments [27]. Even the ligand-based comparative molecular similarity indices analysis (CoMSIA) model (Q^2^ = 0.525, R^2^_ncv_ = 0.891, R^2^_pred_ = 0.807), using 129 reported H_1_R antagonists, had good predictive quality for predicting the bioactivities of new chemicals. Subsequent molecular docking and simulation of these reported antagonists unraveled their binding modes in the active site of H_1_R [64].

### 2.2. Computational Studies of H_2_R and Its Ligands

Although H_2_R is a promising drug target, the computational modeling of H_2_R (UniProt id:P25021, 359 amino acids) has not been studied extensively due to the lack of the crystal structure which hampered the drug discovery efforts. This shortcoming makes development of protein models for structure-based approach a necessity [109]. The quality of the generated homology models largely depends on the selection of template(s) and the sequence alignment while robustness is judged on the basis of their ability to differentiate between known actives and decoys [110,111].

#### 2.2.1. Homology Modeling of H_2_R

Homology modeling that combines multiple templates usually yields better receptor structures for drug discovery processes. Since hH_1_R and hH_2_R have low sequence similarity and identity, the β_1_AR (PDB id:4BVN), hβ_2_AR (PDB id:2RH1), hD_3_R (PDB id:3PBL) and hH_1_R (PDB id:3RZE) were chosen as templates, depending upon TM similarities and identities for H_2_R homology modeling, as for example, in the study of Saxena et al. [66]. Krzan et al. [85] generated a H_2_R homology model employing multiple webservers and programs: I-TASSER [112], MODELLER [113], SWISS MODEL [114] and Pyre2 [115] using the following templates: hH_1_R (PDB id:3RZE), the neurokinin 1-receptor (PDB id:2KS9), human β_2_-adrenergic receptor (PDB id:2RH1), human β_1_-adrenoceptor (PDB id:4BVN), and M_3_ muscarinic acetylcholine receptor (PDB id:4DAJ). The best model was selected using the statistics for non-bonded interactions generated by the ERRAT tool [116] and stereochemical properties obtained from PROCHECK [117]. Chaudhary et al. [118] screened a range of phytochemicals present in *Ficus religiosa* for binding to hH_2_R. For this study a homology model of hH_2_R was generated on the basis of similarity search using four structures of β_1_-adrenergic receptor (PDB ids:2VT4, 2Y00, 4BVN and 5A8E) as templates. In another study, a template of β_1_-AR (PDB id:2VT4) was used for homology modeling and a model quality was assessed by Ramachandran plot while AutoDock was used for docking of compounds [119]. Recently, Boddupally et al. [120] also generated a hH_2_R model based on a β_1_-AR template (PDB id:6H7J) using MODELLER and evaluated it by PROCHECK and Ramachandran plot. The modeling was followed by docking of twenty natural flavonoid compounds to the receptor in AutoDock. In the above three studies only β_1_-AR was used as a template so the modeled structure of H_2_R showed some limitations in model quality.

#### 2.2.2. H_2_R-Targeted Ligands and Their Interactions at H_2_R Active Site

**Figure 5:**
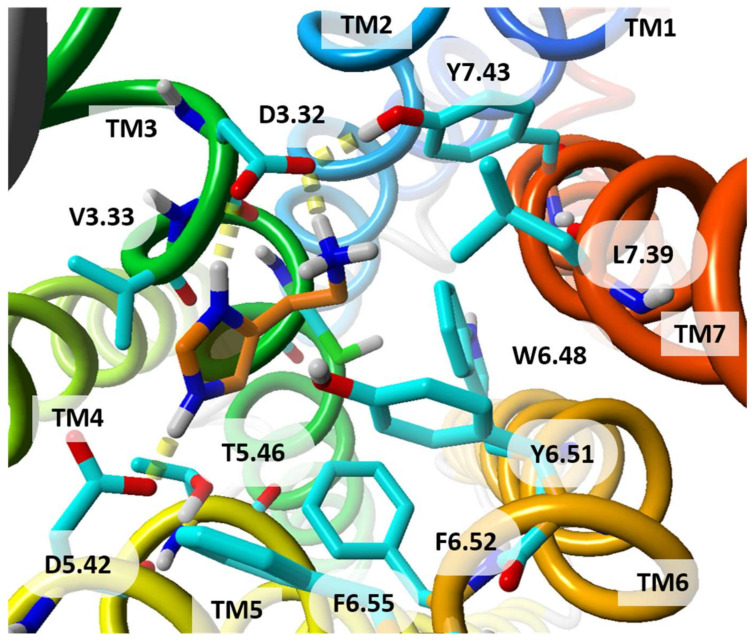
Histamine molecule docked to the homology model of hH_2_R. The histamine is doubly positively charged. Both charged moieties of histamine are bound to D^3.32^. The imidazole ring is additionally bound to D^5.42^. The residue numbers are shown in Ballesteros-Weinstein numbering scheme. Histamine is shown with its carbon atoms colored in orange. View from extracellular side. TM helices are colored from blue (TM1) to red (TM7).

In an effort to design, develop and optimize selective H_2_R as well as dual H_1_R and H_2_R ligands, molecular docking using the Schrodinger package provided insights on how to rationalize the binding of octahydropyrazinopyridoindole class of compounds [66]. Additionally, docking the standard H_2_R antagonists such as metiamide, cimetidine, ranitidine and famotidine to the homology modeled hH_2_R enabled comparison of properties of selective ligands of H_1_ and H_2_ receptors. It has been found that hydrophobic regions are important for selective hH_1_R antagonists whereas polar features for selectivity of hH_2_R antagonists. However, the hydrophobic interaction in vicinity of polar region was also discriminating for hH_2_R ligands due to the presence of V^3.33^ residue in H_2_R instead of Y^3.33^ in H_1_R and other histamine receptors. Details of the orthosteric binding site of homology model of hH_2_R are shown in Figure 5. Analysis of the residue properties further confirmed TM5 to be the most dissimilar region between hH_1_R and hH_2_R, followed by TM6 and TM3. A mutagenesis study revealed that the residue D^3.32^ is vital for histamine and antagonist binding, while D^5.42^ and T^5.46^ are important for the selectivity and kinetics of histamine binding. The presence of V^3.33^ and D^5.42^ in hH_2_R introduces a bulkier space and negatively charged environment at this position as compared to Y^3.33^ and T^5.42^ in hH_1_R, respectively. Other important hydrophobic residues, W^6.48^, Y^6.51^, F^6.52^ and F^6.55^, are the same in both H_1_R and H_2_R. The representative class of hH_2_R antagonists was found to form hydrogen bonds with D^3.32^, D^5.42^, Y^6.51^, and N159^ECL2^ and hydrophobic contacts with the aromatic triad in H_2_R (W^6.48^, Y^6.51^, and F^6.52^). Analysis of the structure-based pharmacophore model for H_1_R and H_2_R indicates that hydrophobic features are important for selective H_1_R antagonism while polar groups are preferable for selective H_2_R antagonism.

Histamine docked to the optimized model of H_2_R asserted the importance of three crucial residues D^3.32^, D^5.42^ and Y^6.51^ for histamine binding. A proton transfer from the charged ethylamino group of histamine to D^3.32^ allowed K175^ECL2^ to undergo large conformational change and approach the D^3.32^ residue. Additionally, from performed quantum-chemical calculations, it was evident that deuteration increased affinity of histamine towards H_2_R binding [85]. Pockes et al. [77] performed studies on dimeric hetarylpropylguanidine-type derivatives which were docked to H_2_R while the monovalent ligands were docked to hH_3_R and hH_4_R. Docking studies were followed by 30 ns MD simulations and the lowest free-energy conformations of compounds formed the strongest H-bond contacts with the residues of the orthosteric binding site: D^3.32^ and D^5.42^ in hH_2_R; D^3.32^ in hH_3_R; and D^3.32^, E^5.46^, E163^ECL2^ and T^5.42^ in hH4R. The residue D^5.42^ accounts for hH_2_R selectivity since it is not present in other histamine subtypes. Different steric effects of residues enclosing the orthosteric binding pocket may be at play since residues V^3.33^, V176^ECL2^-Q177^ECL2^ of hH_2_R are less voluminous when compared to Y^3.33^, F184^ECL2^-Y185^ECL2^ of hH_1_R; Y^3.33^, F192^ECL2^-F193^ECL2^ of hH_3_R; and Y^3.33^, F168^ECL2^-F169^ECL2^ of hH_4_R. During the study, it was observed that dimeric compounds showed better affinity towards hH_2_R while monomeric ligands showed better affinity towards hH_3_R and hH_4_R [77]. A representative of the former ligands is Compound **5** shown in Figure 2.

### 2.3. Computational Studies on H_3_R and Its Ligands

#### 2.3.1. Homology Modeling and Structural Aspects of H_3_R

The homology models of H_3_R (UniProt id:Q9Y5N1, 445 amino acids) were generated using a variety of templates starting from bovine rhodopsin (PDB id:1U19) [59]. In many studies the crystal structure of inactive hH_1_R (PDB id:3RZE) was used as a template for construction of the homology model of inactive hH_3_R since their sequences possess 31.4% identical residues [57,58,79,81]. The homology model of H_3_R was also built using M_3_ muscarinic acetylcholine receptor (PDB id: 4DAJ) [51,62,94,95]. Recently, researchers have described a H_3_R homology model taking the crystal structure of M_2_ muscarinic acetylcholine receptor as a template (PDB id: 3UON) [67,121,122].

Jonczyk et al. [80] generated H_3_R homology models and used a hybrid assessment of these models based on knowledge-based scoring algorithm and two-step docking protocols including GOLD and Glide. The models also passed the quality analysis performed using BCL::Score, QMEAN and PSVS methods. A model built on the M_3_R template was preferred as compared to H_1_R or models built on mixed template alignments—they were characterized by significant differences in the most conformationally diversified ECL2 loop. Most models generated by MODELLER proved to be much better that those of SwissModel, I-TASSER and Jackal. In the best H_3_R model, 3-7 lock switch was considered as the interaction between the side chains D^3.32^ and W^7.43^, whose breakup can promote receptor activation. Additionally, the perpendicular position of residue W^6.48^, with respect to the helix axis forced by L^7.42^, favored recognition of H_3_R-specific ligands and interaction with E^5.46^. Inactive conformation of H_3_R is maintained by residues L^2.43^, L^2.46^, I^3.43^, I^3.46^ and I^6.40^ in the center of the receptor creating a hydrophobic barrier inside the receptor as in other GPCRs. Breakdown of this barrier is an important step in receptor activation and opens a gate for a continuous intrinsic water pathway [123,124].

The multiple template approach has also been applied for H_3_R homology modeling by using H_1_R (PDB id:3RZE), M_2_R (PDB id:3UON) and M_3_R (PDB id:4U15) as templates while ECL2 was built on M_2_R and M_3_R. The models were ranked for possessing a crucial ionic protein-ligand interaction with residue D^3.32^ considered essential for ligand binding. It was also found that a conformation of residue E^5.46^ was more advantageous for ligand binding when it was pointing toward the binding pocket [52]. Another multiple template approach was employed by Hauwert et al. [60] for modeling H_3_R using H_1_R (PDB id:3RZE), M_3_R (PDB id:4U15), dopamine D_3_R (PDB id:3PBL), serotonin 5-HT_1B_R (PDB id:4IAR) and serotonin 5-HT_2B_R (PDB id:4IB4) receptors as templates and the MODELLER program.

#### 2.3.2. Ligands Targeting H_3_R and Their Interactions

**Figure 6:**
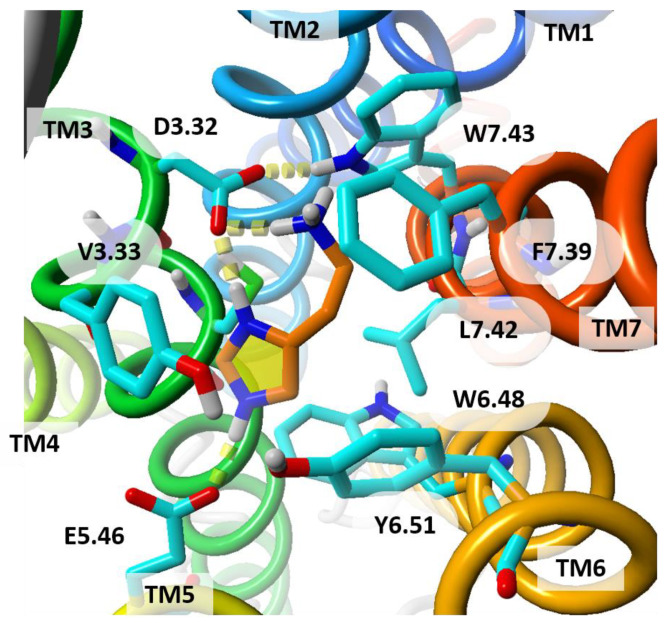
Histamine molecule docked to the homology model of hH_3_R. The histamine is doubly positively charged. Both charged moieties of histamine are bound to D^3.32^. The imidazole ring is additionally bound to E^5.46^. The histamine hydrophobic linker is exposed toward hydrophobic residues F^7.39^ and L^7.42^. The residue numbers are shown in Ballesteros-Weinstein numbering scheme. Histamine is shown with its carbon atoms colored in orange. View from extracellular side. TM helices are colored from blue (TM1) to red (TM7).

Like other GPCRs H_3_R is sodium sensitive, as it was made evident by using a mathematical model and MD simulations. It was also calculated that an H_3_R inverse agonist thioperamide binds to the orthosteric binding site of hH_3_R preferentially in a presence of Na^+^. In the presence of Na^+^, the positively charged imidazole moiety of thioperamide is located “above” the highly conserved residue D^3.32^ while in its absence the same moiety is located “below” D^3.32^ and directed toward the highly conserved D^2.50^, which forms the allosteric site for binding a sodium ion [58]. Thioperamide spans horizontally between two negatively charged residues D^3.32^ and E^5.46^ and the ligand is embedded in a pocket between helices TM3, TM5 and TM6 [58,59]. However, in a study [80] based on another template, M3 muscarinic receptor, thioperamide was docked vertically and interacted only with D^3.32^. There are two negatively charged residues, D^3.32^ and E^5.46^, in the orthosteric ligand binding pocket of hH_3_R (similarly to hH_4_R). The former residue can bind to the protonated amine and is essential for interactions with agonists, including histamine. The second acidic binding point is created by E^5.46^ and adjacent tyrosine Y^6.51^. In a study by Jonczyk et al. [80], a series of amine antagonists including JNJ5207852 was docked to hH_3_R in vertical poses, and the protonated amines of these ligands were bound to E^5.46^, Y^3.33^ and Y^6.51^. It was also found that antagonist clobenpropit with protonated imidazole ring and isothiourea fragments, used both acidic points, D^3.32^ and E^5.46^, in the ligand binding space: imidazole interacted with D^3.32^ while isothiourea group, as a second protonated system, created a salt bridge with E^5.46^. It is in contrast to results obtained by Kim et al. [125] which employed a model of hH_3_R based on the human β_2_-adrenergic receptor. Doubly charged clobenpropit was bound to D^3.32^ by its isothiourea group while the imidazole ring was bound to E^5.46^ and adjacent residues. Although it cannot be excluded that both poses are allowed, the future experimentally determined structures of histamine receptors with ligands will help docking studies enormously. Details of the orthosteric binding site of homology model of hH_3_R are shown in Figure 6.

Binding of more complex antagonists of H_3_R was possible by extending the ligands toward the external allosteric site residues, R^6.58^, Y^7.35^ and residues of the ECL2 loop (Y189, A190, F193 and Y194) as documented by Kumar et al. [62] for novel 4-aryl-6-methyl-5,6,7,8-tetrahydroquinazolinamines as anti-obesity candidate drugs. In the study performed by the Gloriam group [50] the pharmacophores were constructed based on the residue-ligand fragments from the GPCR crystal structures. Thanks to the chemogenomic techniques that detect local similarities within the transmembrane binding pocket the whole scaffolds or ligands can be exchanged between targets. Performing such binding pocket comparisons is reasonable since many high-quality GPCR crystal structures with ligands are currently known. Using the above methodology for the fragment-based prospective virtual screening employing pharmacophores and molecular docking methodology they identified five novel neutral antagonists and one inverse agonist which were later confirmed in G_q_-coupled pharmacological assay. They also found that residue E^5.46^ contributes to selectivity in H_3_R and H_4_R as compared to H_1_Rs/H_2_Rs while residues at positions 2.64, 2.65, 5.42, 6.52, 6.55, and 7.36 confer H_3_R/H_4_R selectivity [50].

Diverse screening protocols were applied for discovery of H_3_R targeted ligands including ligand-based approaches exploiting the similarities with respect to known actives as well as structure-based approaches exploiting the 3D-chemical interactions between ligands and the target structure [126]. Ghamari et al. [127] employed individual and combined hybrid structural similarity (FP2 fingerprint in 2D search, and also Electroshape and Spectrophores methods in 3D search) and pharmacophore-based approaches using the ZINCPharmer [128] and ZINC15 database followed by lead optimization, and identified potential drug-like anti-H_3_R ligands in micromolar and submicromolar activity range (including Compound **7** in Figure 2). The geometric center of residues D^3.32^, T^3.37^, Y189^ECL2^, F^5.38^, E^5.46^, W^6.48^, and Y^6.51^ was set as the center of the binding site. Docking of pitolisant and other ligands was performed with two distance constraints with E^5.46^ and Y189^ECL2^ in GOLD. The phenyl ring of pitolisant was found sandwiched between Y189^ECL2^ and F^7.39^ while piperidine nitrogen formed an ionic bond with E^5.46^ and a H-bond with Y^3.33^. Four-featured 3D-pharmacophore model comprising two hydrophobic regions, a positively ionizable moiety, and a hydrogen bond acceptor was generated from a docked pitolisant-H_3_R complex using LigandScout [129]. The obtained hits possessed a common scaffold containing a basic moiety and an aromatic/hydrophobic moiety joined by a linker. The basic moiety interacted with E^5.46^, the linker interacted with Y^3.33^ and Y^6.51^, while the hydrophobic/aromatic moiety was observed to interact with Y189^ECL2^ and F^7.^^39^ [51,127]. In another study a quantitative structure-activity relationship (QSAR) model, employing a genetic algorithm coupled with partial least square and stepwise multiple linear regression methods, was used with two major descriptors: connectivity information and mean absolute charge in predicting H_3_R antagonistic activity. The additional molecular docking revealed the crucial role of residues Y^3.33^, Y189^ECL2^, F193^ECL2^, L^5.39^, E^5.46^, W^6.48^, Y^6.51^, M^6.55^, Y^7.35^ and F^7.39^ in the interaction with H_3_R targeted ligands [91]. Schaller et al. [52] used a ligand-guided homology modeling strategy that resulted in identification of two H_3_R ligands with nanomolar affinity, including Compound **8** (Z27743747) in Figure 2. Pharmacophore models were generated by taking nine reported H_3_R antagonists and the virtual screening was performed using the Enamine library. The high-quality model of H_3_R was used for docking while keeping constraints focused on interactions with D^3.32^ and E^5.46^. The obtained hits interacted with D^3.32^ and Y^3.33^ as well as with several other hydrophobic residues. The additional π-cation interaction with F^7.39^ was predicted to contribute to the superior H_3_R affinity of ligands [52].

The H_3_R antagonists have been classified as imidazole- and non-imidazole-based compounds. Preliminary studies have focused on the development of the imidazole type compounds which are structurally similar to histamine but, due to some undesirable features, they failed to enter the pharmaceutical market [130]. Hence, researchers turned their attention to imidazole ring bioisosteres in an attempt to develop non-imidazole type compounds such as piperidine, piperazine and pyrimidine. This approach resulted in e.g., pitolisant. The ligands with diverse scaffolds have been docked to H_3_R models, including amino acetylenic benzophenone derivatives [59], flavones [79], isoflavones [90], *tert*-amylphenoxyalkyl (homo)piperidine derivatives [88], piperazine derivatives including tert-butyl/tert-pentylphenoxyalkyl, acetyl/propionyl phenoxyalkyl, 4-pyridyl, and (dihydro)benzofuranyl [29,67,86,89], tetrahydroquinazolinamines [62] or pramepexole carbamodithiolate metal complexes [131]. A series of flavone derivatives was designed and docked to H_3_R model. The flavones were observed to form a salt bridge with E^5.46^, π-anion bond with D^3.32^, H-bond with Y^6.51^, and the hydrophobic interactions with Y^3.33^, C^3.36^, A^5.42^, W^6.48^, Y^6.51^, and L^7.42^ [79] while isoflavones formed a salt bridge with E^5.42^ and π–π interactions with Y^3.33^ and F^5.38^ [90]. In another study, the *tert*-amylphenoxyalkyl (homo)piperidine derivatives were docked to H_3_R model built on M_3_ muscarinic acetylcholine receptor in GPCRM structure modeling server [132]. It was observed while docking these derivatives that a salt bridge was formed with residue E^5.46^, and π–π stacking with Y^3.33^, F^5.39^ and/or Y189^ECL2^ [88]. In the case of a novel series of piperazine derivatives (tert-butyl/tert-pentylphenoxyalkyl, acetyl/propionyl phenoxyalkyl and 4-pyridyl), the ligands were observed to form H-bond/salt bridge with E^5.46^, and H-bond with Y^6.51^ while forming π–π interactions with W^6.48^ and F^5.47^. Additionally, the ligands were found to be stabilized through π–π stacking with at least one of the following residues: Y^3.33^, Y^7.35^ and F193^ECL2^ [67]. An additional hydrogen bond with residue R^6.58^ could have a positive influence on H_3_R affinity [89]. Ligands with 4-pyridyl piperazine scaffold were found to be H_3_R selective antagonists as compared to H_3_R, D_2_R, M_1_R and α_1_-adrenergic receptors [61]. Dihydrobenzofuranyl-piperazines analogs with 5-phenyl and allylpiperazine modifications were recently found to be the potent hH_3_R antagonists with nanomolar affinity [86].

Wagner et al. [81] have evaluated a series of 4-(3-aminoazetidin-1-yl)pyrimidin-2-amines as high affinity H_3_R partial agonists obtained from an in-house screening campaign (Compound **11**). All these ligands along with histamine, when docked using PLANTS and after MD simulations as H_3_R complex, have maintained stable crucial interactions with D^3.32^, E^5.46^ and Y^6.51^ [81]. Two bidirectional GPCR photoswitchable antagonists with substituted azobenzene scaffold (VUF14738 and VUF14862 (Compound **9** in Figure 2) were developed by Hauwert et al. They possessed 10–100 fold higher H_3_R selectivity over H_1_R and no measurable H_4_R affinity [60]. Recently, Z3303614736 have been found to be new strong inverse agonist of H_3_R that inactivates the receptor in a nanomolar concentration range. It was identified by using the intramolecular H_3_R biosensor based on bioluminescence resonance energy transfer (BRET) that is sensitive to the conformational dynamics of H_3_R and can be used to screen for the receptor agonists and inverse agonists in a live cell [133]. Nonimidazole antagonists/inverse agonists of H_3_R with nanomolar activity as anticonvulsant drugs were obtained by Song et al. [134] by linking the H_3_R pharmacophore (aliphatic amine with propyloxy chain) with the 1,2,4-triazole moiety. Docking studies indicated that these ligands were bound to Y^3.33^ and E^5.46^, and additionally to R^6.58^, F193^ECL2^ and M^6.55^ [134].

#### 2.3.3. Multi-Target H_3_R Ligands

Recently, the multi-target-directed ligands of histamine receptors gained a lot of attention [45,57,68,78,94,95] that led to development of multi-targeted ligands of H_3_R, serotonin receptors, as well as acetylcholinesterases (AChE and BuChE), for potential treatment of neurodegenerative diseases such as Alzheimer’s disease. Jonczyk et al. [68] identified multi-targeted piperazine derivatives to be blockers of AChE and BuChE as well as of hH_3_R. The ligands were bound to E^5.46^, as this was suggested to be a crucial interaction for H_3_R antagonistic activity, while the other interactions were with residues F163^ECL2^, Y^3.33^ and Y^6.51^ [68]. Lepailleur et al. [49] performed ligand-based pharmacophore-guided virtual screening of CERMN chemical compound library (17,194 compounds) using six active H_3_R ligands with the purpose of designing dual targeting H_3_R/5-HT_4_R ligands. Ligand docking was performed using the Schrodinger Glide Induced Fit procedure. The best ligands appeared to be benzo[*h*] [1,6] naphthyridine derivatives. The pharmacophoric features correlated well with the interacting residues: protonated amine (basic center) formed a salt bridge with D^3.32^, the aromatic system formed a π−π interaction with indole ring of W^6.48^, the hydrophobic part was surrounded by residues L^3.29^, W^3.28^ and F^7.39^, and the NH of the tricyclic system served as a H-bond donor bound to the side chain of E^5.46^. The ligands including the most potent Compound **6** in Figure 2 possessed two positively ionizable groups (imidazole, alkylamine) that were sometimes supposed to interact with residues D^2.50^ or with E175^ECL2^ and E191^ECL2^ [49]. Darras et al. [57] performed computational studies followed by synthesis and biological evaluation for synergistic effects of tri- and tetracyclic nitrogen bridgehead compounds reported as dual acting hH_3_R antagonists and AChE inhibitors. The tricyclic partial structure of the most potent and selective H_3_R compound in this study remained stably bound in a pocket formed by residues L^3.29^, Y^3.33^, C^3.36^, W^6.48^, Y^6.51^, M^6.55^, and F193^ECL2^. Two stable ionic interactions between D^3.32^ and a positively charged piperidine moiety of compounds, as well as between E^5.46^ and a positively charged amidine moiety were observed during entire MD simulation. Also, an aromatic interaction was observed between an aromatic moiety of a docked Compound **10** with Y^3.33^ and F193^ECL2^ [57].

A series of twenty new chlorophenoxyalkylamine derivatives was reported as dual acting hH_3_R antagonists and AChE/BuChE inhibitors [94,95]. The hH_3_R model was developed using GPCRM webserver and validated using pitolisant as a reference H_3_R ligand. All these compounds were docked to target proteins using Schrodinger Glide software. It was observed that irrespective of alkyl chain length, chloro substituted phenyl rings were in close contact with ECL2 residues (W174, L177, A190, E191, Y194 and W196) [94,95]. The multitarget-directed ligands with H_3_R antagonistic activity coupled with the ability to inhibit acetyl/butyrylcholinesterases and monoamine oxidases A/B, potentially suitable for the treatment of Alzheimer’s or Parkinson’s disease, were studied by Bautista-Aguilera et al. [78]. All the studied compounds revealed an interesting neuroprotection profile against oligomycin A, okadaic acid (as a model of the hyperphosphorylation of tau), and β-amyloid peptide Aβ_25–35_. Of all ligands the non-imidazole ligand, contilisant, had the best properties. Recently, 26 non-imidazole histamine H_3_R ligands and 23 xanthone derivatives, rationally designed using a pharmacophore model for H_3_R antagonists/inverse agonists, have been confirmed as potential anti-Alzheimer agents by docking to H_3_R and acetyl- and butyrylcholinesterases. The most promising derivatives combined the flavone moiety via a six carbon atom linker with a heterocyclic moiety, such as azepane, piperidine or 3-methylpiperidine [121,122].

### 2.4. Computational Studies on H_4_R and Its Ligands

#### 2.4.1. Homology Modeling and Structural Aspects of H_4_R

H_4_R (UniProt id:Q9H3N8, 390 amino acids) possess 40% identity in TM region to H_1_R which led many researchers to generate H_4_R homology models using H_1_R crystal structure as a template. However, there are substantial differences in their binding sites as the residues K^5.39^, N^5.46^ and G^7.42^ in H_1_R are equivalent to L^5.39^, E^5.46^ and Q^7.42^ in H_4_R. The large sequence similarity (37%) and structural similarity (58%) in the binding site between H_3_R and H_4_R, confirmed by mutagenesis studies, emphasized a crucial role of identical residues: D^3.32^, E^5.46^, Y^3.33^, W^6.48^ and Y^6.51^ in ligand binding that results in similar ligand poses in the binding site and can explain the mechanism of dual H_3_R/H_4_R antagonism [36,57]. Importance of specific residues for ligand binding was confirmed by mutations: E^5.46^Q had decreased the affinity of clobenpropit and its derivatives toward H_3_R and H_4_R while mutations L^5.39^V and E^5.46^Q decreased the affinity of ligands against H_4_R. The mutagenesis also revealed importance of N^4.57^, L^5.39^ and E^5.46^ for antagonist binding while featuring H-bonds/electrostatic interactions with two negatively charged residues D^3.32^ and E^5.46^ to be important for receptor activation [27,53,72,135]. Combined mutagenesis and protein-ligand modeling studies performed to explain H_3_R and H_4_R selectivity indicate that the residue at position 4.57 (Y^4.57^ in H_3_R and N^4.57^ in H_4_R) is directed toward the ligand binding pocket. This suggests that the TM4 of H_3_R and H_4_R remains undistorted making the constriction unique for the H_1_R subtype [136]. The pharmacological profile of H_4_R ligands was recently reviewed by our group [137].

The residues in ECL2 (the largest and the most structurally diverse extracellular loop of class A GPCRs) are also important for binding of ligands to all monoaminergic GPCRs as revealed by site-directed mutagenesis experiments. ECL2 also plays an important role as a stabilizer of the inactive state of the receptor [138]. Mutagenesis of two ECL2 phenylalanine residues (FF motif) in hH_4_R resulted in reduced constitutive and ligand-induced receptor activation, which points to a significant involvement of this motif in receptor activation [80,82,96]. Mutations of these residues can change the pharmacological properties of ligands: thioperamide is a partial inverse agonist at hH_4_R F169^ECL2^V, which is a mutant with reduced constitutive activity, and a neutral antagonist at hH_4_R F168^ECL2^A a mutant devoid of constitutive activity. It was concluded that F168^ECL2^ is a key determinant of H_4_R constitutive activity, ligand binding and potency as compared to F169^ECL2^. These studies reveal a crucial role of the FF motif, F168^ECL2^ and F169^ECL2^, in both ligand-receptor interactions and constitutive activity (interconversion between active and inactive conformation) of the wild-type hH_4_R [82].

#### 2.4.2. Ligands Targeting H_4_R and Their Interactions

**Figure 7:**
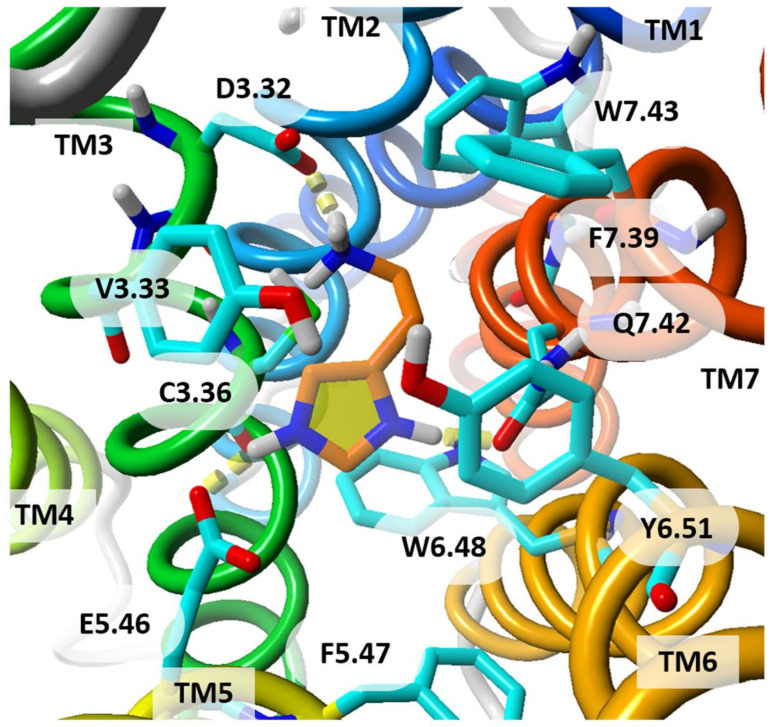
Histamine molecule docked to the homology model of hH_4_R. The histamine is doubly positively charged. The amine moiety of histamine is bound to D^3.32^ whereas the imidazole ring is bound to E^5.46^ and Q^7.42^. The histamine hydrophobic linker is exposed toward hydrophobic residues F^7.39^ and inversely rotated W^7.43^. The residue numbers are shown in Ballesteros-Weinstein numbering scheme. Histamine is shown with its carbon atoms colored in orange. View from extracellular side. TM helices are colored from blue (TM1) to red (TM7).

Kiss et al. identified novel histamine H_4_R ligands through the ensemble docking based on homology model conformers obtained from MD simulations. Such representative hH_4_R conformers were found to be more suitable for the identification of H_4_R antagonists than the initial homology models [25]. It was also found that X-ray and homology model structures may be complementary, or at least able to sample different protein conformations leading to non-overlapping hits and can provide important starting points for fragment-based lead discovery for other GPCRs [6]. Labeeuw et al. [22] discovered a potent antagonist (Compound **12**) among 2-benzothiazolylphenylmethyl ether analogues. It was identified in a virtual screening of a corporate compound collection based on H_4_R model, followed by hit optimization with the purpose of designing potent and selective H_4_R antagonists. As for the binding mode the imidazole ring of the scaffold was suggested to form a salt bridge with D^3.32^, the oxygen of the linker a H-bond with the phenol moiety of Y^3.33^, and the nitrogen atom of the benzothiazolyl group a H-bond with Y^6.51^. Istyastono et al. [53] have explored complementary retrospective and prospective SBVS using β_2_R-based and H_1_R-based H_4_R homology models with a IFP scoring on fragment-like ligands, which allowed identification of H_4_R ligands (including Compound **14**) that were not spotted during LBVS runs. Although the authors observed higher enrichments in a model based on β_2_AR as compared to a model based on H_1_R, an exchange of templates did not significantly affect SBVS accuracy. The TM binding pocket was very similar in both H_4_R models, based on β_2_AR and H_1_R, with little differences in ECL2. In the β_2_AR-based H_4_R model, the chlorine atom of JNJ7777120 was accommodated between L^5.39^, T^6.55^ and F168^ECL2^, while in the H_1_R-based model it was located close to residues L^5.39^, T^5.42^ and F168^ECL2^ [53]. Details of the orthosteric binding site of homology model of hH_4_R are shown in Figure 7.

With the constantly growing number of H_4_R ligands, the applicability of a large group of azine derivatives has been increased. A promising series of novel selective H_4_R antagonists, such as 2-amino-4-methylpiperazine-1,3,5-triazines, was designed and synthesized by different research groups by introducing variously substituted arylethenyl moieties [24,92,139]. Lazewska et al. [24] docked these molecules on the validated H_4_R homology model built using a template of H_1_R. All compounds with triazine core including Compound **15** were found to interact with D^3.32^ in two possible ligand orientations in the binding pocket of H_4_R. The protonated nitrogen of the most active ligands formed a weak interaction with E^5.46^. The triazine core of ligands was found to interact with Y^3.33^ and also with T^5.42^ and Y^6.51^. Halogen atoms *para* substituted to the benzene ring were found to fit in the hydrophobic pocket formed by residues V^2.53^, I^2.58^ and W^3.28^. Among them, 4-(cyclohexylmethyl)-6-(4-methylpiperazin-1-yl)-1,3,5-triazin-2-amine exhibited the highest hH_4_R affinity with K_i_ of 160 nM and showed anti-inflammatory properties in the carrageenan-induced edema test during preliminary studies in mice [24]. Mogilski et al. [139] identified two promising structures with chlorine and bromine atoms at *para*-position in the aromatic ring as potential anti-inflammatory agents more potent than the reference compound JNJ7777120. They were shown to inhibit the inflammatory response in two different in vivo models of inflammation, the carrageenan-induced model and zymosan-induced peritonitis [139].

Levoin et al. [23] discovered novel scaffolds using virtual screening on the Prestwick library in a two-step process: (i) using a “scout screening” methodology for a small size chemical library with the very diverse structures, and (ii) by using the refined 3D model of H_4_R to conduct a widened virtual screening. This two-step strategy proved to be successful, both in terms of structural diversity and hit rate (23%). Moreover, the hits had the high affinities for H_4_R, with the most potent ligands in the nanomolar affinity range [23]. Ko et al. discovered a novel series of pyrido[2,3-*e*]tetrazolo[1,5-*a*]pyrazine analogues as orally available potent and highly selective H_4_R antagonists with strong antipruritus and anti-inflammatory activity [26]. The authors used a pharmacophore-based virtual screening on ZINC12 database incorporating iterative clustering with Tanimoto similarity (similarity coefficient ≥0.9). This approach resulted in eight pharmacophores with four features. A H_4_R model was constructed using the H_1_R template and refined using MD simulations. The basic amine of *N*-methylpiperazine of a lead formed a crucial ionic interaction with D^3.32^ while the main scaffold formed the π−π interactions with residues Y^3.33^, W^6.48^, Y^6.51^, and F168^ECL2^.

Geyer et al. [63] studied constrained analogues of the imidazolylbutylcyanoguanidines substituted by cyclopentane-1,3-diyl moiety and identified the most active enantiomer (Compound **13**). The imidazole moiety of enantiomers of this compound was found to be embedded in a slightly different orientation in the binding pocket of H_4_R, which resulted in an order of magnitude higher affinity of Compound **13** to H_4_R, in comparison to other enantiomers, and in two orders of magnitude higher selectivity compared to H_3_R. Such differences originated from different interactions at residues 6.52 and 7.42 in both receptors. The imidazole ring and the cyanoguanidine group were in contact with these residues in H_4_R (S^6.52^ and Q^7.42^) but not in H_3_R (T^6.52^ and L^7.42^) [63]. Hammer et al. [31] studied the substituted 2,4-diaminopyrimidines as dual ligands of H_1_R and H_4_R and their docking poses were stabilized in MD simulations. In this study, the positively charged piperazine moiety was observed to interact with D^3.32^ while Y^6.51^ formed a stable interaction with E^5.46^. The authors speculated that the subtype differences between H_1_R and H_4_R, hampering the identification of dual, high affinity H_1_R/H_4_R ligands, might be due to the residue at position 3.40 (I^3.40^ in H_1_R and V^3.40^ in H_4_R) [31]. Additionally, the residue at position 7.42 (L^7.42^ in H_3_R and Q^7.42^ in H_4_R) might be important for H_3_R/H_4_R selectivity as it was shown in a recent study by Correa et al. for benzofuranyl-piperazine with a carbonyl group in a linker between these moieties [86].

## 3. Conclusions

The ligands of histamine receptors are increasingly used in treatment of various pathological conditions including allergic diseases, inflammation, neurological disorders, and possibly also obesity. Three histamine receptors, H_1_R-H_3_R, have been well established while H_4_R is a novel, attractive drug target for allergic and inflammatory disorders. HR antagonists proved to be efficacious and relatively safe in animal models and several clinical trials are currently conducted or have been recently completed. Extensive drug design and medicinal chemistry attempts exploring structure-activity relationships led to the development of numerous novel selective HR ligands. Molecular docking proved to be a valuable technique to analyze ligand recognition and together with virtual screening has led to important drug discoveries in the field of histamine receptors. However, more crystal structures of all histamine receptors, both in inactive and active states, are needed to profoundly understand the structural details of their binding sites and the resulting signaling in order to design specific ligands with desired pharmacological profiles.

## Figures and Tables

**Table 1:** Overview of the drug design strategies for discovery of HR ligands. In parenthesis the PDB ids of templates used in homology modeling.

Receptor	Templates	Receptor State	Strategy	Hit Rate	Reference
H_1_R	β_2_-adrenergic (2R4R)	Inactive	SBVS of Phytochemical inhibitors	5 hits	[48]
H_1_R	3RZE	Inactive	IFP-based SBVS	60.6% (20/33)	[9]
			PLANTS-based SBVS	45.5% (15/33)	
			Combined IFP and PLANTS scoring based SBVS	73.1% (19/26)	
H_3_R	H_1_R (3RZE)	Inactive	Pharmacophore-based virtual screening	100% (5/5)	[49]
H_3_R	H_1_R (3RZE)	Inactive	Prospective crystal structure-based pharmacophore virtual screening	8% (6/76)	[50]
H_3_R	M_3_ muscarinic receptor (4DAJ)	Inactive	FP2 fingerprint/Electroshape / Spectrophores/LBVS	50% (2/4)	[51]
			Hybrid VS	100% (1/1)	
H_3_R	H_1_R (3RZE), M_2_ muscarinic (3UON), M_3_ muscarinic (4U15)	Inactive	Pharmacophore screening and redocking.	25% (2/8)	[52]
H_4_R	H_1_R (3RZE)	Inactive	Single Structure	22% (11/50)	[6]
			Ensemble Docking	16% (8/50)	
			Overlap hits	27% (4/15)	
H_4_R	H_1_R (3RZE)	Inactive	Ligand-based chemoinformatics: Intelligent Learning Engine/Iterative Stochastic Elimination/Extended connectivity fingerprint (ECFP4).	11 hits	[12]
H_4_R	Bovine rhodopsin (1F88)	Inactive	Ensemble docking	5.3% (4/75)	[25]
			Ensemble docking followed by consensus scoring.	15.4% (2/13)	
H_4_R	β_2_R (2RH1)	Inactive	Prospective SBVS.	26% (6/23)	[53]
	H_1_R (3RZE)	Inactive	Prospective SBVS.	21.4% (3/14)	
H_4_R	Bovine rhodopsin (1L9H)	Inactive	Homology model refined by “scout screening”, VS using ECFP_4 fingerprint.	23% (28/120)	[22,23]
H_4_R	H_1_R (3RZE)	Inactive	Pharmacophore-based virtual screening (Tanimoto similarity coefficient ≥0.9).	1% (3/291)	[26]

**Table 2:** Residues (in B-W numbering scheme) of HR subtypes involved in the binding of ligands studied in particular papers.

Residue	Residues				Reference
	H_1_R	H_2_R	H_3_R	H_4_R	
2.50			D80		[49,54]
2.61	N84	S75	Y91	Y72	[26,55,56,57,58,59,60,61,62,63,64]
2.62	I85				[31]
2.64	Y87	Y78	Y94	H75	[55,60,63]
2.65	L88				[55]
ECL1	W93				[65]
3.28	W103	Y94	W110	W90	[54,55,59,62,63,66,67,68]
3.29	L104	T95	L111		[55,57,62,66]
3.32	D107	D98	D114	D94	[6,9,12,22,23,24,25,26,31,48,49,51,52,53,54,55,56,57,58,59,60,62,63,64,66,69,70,71,72,73,74,75,76,77,78,79,80,81,82,83,84,85,86]
3.33	Y108	V99	Y115	Y95	[6,12,22,24,26,31,51,52,55,56,57,59,60,62,63,64,66,68,69,70,71,72,75,76,77,78,79,80,82,83,84,86,87,88,89,90,91,92]
3.36	S111	C102	C118	C98	[6,12,31,48,57,64,66,76,79,88,90]
3.37	T112		T119	T99	[12,31,51,57,64,73,78,88]
3.40	I115	I106	A122	V102	[31,55,57,64,76,78,88,89]
3.41	F116				[31]
4.56	W158	L149	L166	V146	[9,31,52,55,56,57,63,64,66,72,73,76,78,90]
4.57			Y167	N147	[6,12,53,93]
4.60		S153		M150	[6,12,77]
4.61		I154			[66]
ECL2		N159	E175		[49,66]
ECL2	H167				[55]
ECL2	R175				[65]
ECL2	R176				[55,65,69]
ECL2		T171			[77]
ECL2	D178			S162	[12,55,83]
ECL2	K179		H187	E163	[12,55,56,58,59,65,66,69,77,82,83]
ECL2	C180	C174		C164	[12,55,66]
ECL2	E181	K175	Y189	E165	[12,51,55,66,67,68,80,85,88,91,94]
ECL2	T182	V176	A190	P166	[12,48,55,62,66,77,80,94,95]
ECL2	D183	Q177	E191		[48,49,54,70,77,94,95]
ECL2	F184	V178	F192	F168	[6,12,26,31,53,54,66,77,78,80,82,96]
ECL2	Y185		F193	F169	[12,31,55,57,58,59,62,68,69,77,80,82,83,89]
ECL2			Y194		[62,94,95]
ECL2			W196		[62,94,95]
5.38	F190	Y182	F198	I174	[6,12,31,51,57,59,63,66,88,90,94]
5.39	K191	G183	L199	L175	[6,12,26,31,53,55,56,57,62,63,66,69,70,71,87]
5.42	T194	D186	A202	T178	[6,12,56,57,63,64,66,75,77,79,82,85,87,90]
5.43	A195	G187	S203	S179	[12,26,31,55,57,63,64,66,82,83,87]
5.46	N198	T190	E206	E182	[6,12,23,24,25,26,31,49,50,51,52,53,57,58,59,60,61,63,64,65,66,67,68,69,75,76,77,79,80,81,82,83,85,86,87,88,89,90,91,92,93,94,95]
5.47	F199		F207	F183	[9,12,26,31,55,64,67,72,76,90]
6.44	F424		F367	V184	[66,72,76,82,90]
6.48	W428	W247	W371	W316	[9,12,24,26,31,49,51,52,53,55,56,57,58,61,63,66,67,72,73,75,76,79,80,84,87,89,90,91,94,97]
6.51	Y431	Y250	Y374	Y319	[6,12,22,24,31,48,49,51,52,53,55,56,57,59,60,61,63,65,66,67,69,70,71,72,73,75,76,77,79,80,81,82,83,85,86,87,88,89,91,92,94,95,98]
6.52	F432	F251	T375	S320	[9,12,31,55,56,63,66,72,73,75,76,83,84,87]
6.55	F435	F254	M378	T323	[9,12,31,53,55,56,57,66,72,75,76,83,84]
6.58	I438		R381	L326	[12,55,61,62,65,80,89]
6.59	A439			S327	[12,55]
ECL3	K442			S330	[12,55]
ECL3	N443			S331	[12,55,65,69,83]
7.35	H450	E270	Y394	Y340	[48,55,56,60,62,67,68,69,73,74,77,83,89]
7.39	I454	L274	F398	F344	[6,23,24,51,52,53,55,57,59,60,62,63,66,68,72,76,84]
7.42	G457	G277	L401	Q347	[6,23,24,26,31,52,53,57,58,63,79,80,82,86]
7.43	Y458	Y278	W402	W348	[23,24,26,31,54,55,56,58,63,66,71,72,80,87]

## Data Availability

Not applicable.

## Abbreviations

GPCRs—G Protein-Coupled Receptors; HRs—Histamine Receptors; TM—Transmembrane; ECL—Extracellular Loop; SBVS—Structure-Based Virtual Screening; LBVS—Ligand-Based Virtual Screening; IFP—Interaction Fingerprint; SAR—Structure-Activity Relationship; MD—Molecular Dynamics.
